# Facial emotion recognition accuracy in women with symptoms of polycystic ovary syndrome: Reduced fear and disgust perception

**DOI:** 10.1177/17455057251359761

**Published:** 2025-07-28

**Authors:** Shree Smruthi Venkateshan, Kirsten A. Oinonen

**Affiliations:** 1 Department of Psychology, Lakehead University, Thunder Bay, Ontario, Canada

**Keywords:** polycystic ovary, emotion recognition, social cognition, women’s mental health, fear detection, androgens

## Abstract

**Background:**

Research suggests that women with polycystic ovary syndrome (PCOS) are more likely to suffer from mental health disorders, emotional distress, and have altered hormone profiles (e.g., higher androgens). Past research suggests facial emotion processing is affected by hormones (e.g., androgens), mental health-related disorders, and may be altered in PCOS.

**Objectives:**

The present study examined whether facial emotion recognition (FER) differs between women with and without PCOS symptoms.

**Design:**

Observational case-control design.

**Methods:**

Three groups of participants (women with provisional PCOS, women without PCOS, and men; *N* = 178) completed a FER task that involved identifying emotions (anger, disgust, fear, happiness, sadness, surprise, or neutral) in images of emotional faces. Overall emotion recognition and emotion-specific accuracy were examined. PCOS symptom severity and provisional diagnoses were also assessed in women via self-report measures, including the polycystic ovary syndrome questionnaire.

**Results:**

Women with provisional PCOS had significantly lower emotion recognition accuracy than those without PCOS, and emotion-specific differences were found for fear and disgust. A significant linear effect also emerged for overall FER, revealing men as the least accurate, followed by women with provisional PCOS, and then women without PCOS.

**Conclusions:**

The results suggest that women with PCOS may have difficulty with emotion recognition, especially fear and disgust. The sex difference in emotion recognition was in line with previous research. These findings are consistent with the theory that androgens affect emotion recognition and suggest implications for PCOS symptoms on women’s emotional well-being and socioemotional functioning.

## Plain language summary

Women with symptoms of polycystic ovary syndrome (PCOS) were less accurate at identifying facial emotions

Accurately recognizing facial emotional expressions affects our ability to develop relationships, to avoid dangerous situations, and contributes to social and occupational success. Women have a subtle advantage over men in their ability to accurately recognize facial emotions. However, little research has looked at differences within women. There are a few theories about what might affect the ability to accurately recognize facial emotions. One theory is that higher levels of androgen-related hormones, such as testosterone, reduce the ability to recognize emotional expressions. This explanation makes sense as men have higher androgens and are less accurate at recognizing facial emotions than women. Our study examined emotion recognition in women reporting symptoms of polycystic ovary syndrome (PCOS). This syndrome affects 4 to 20% of women of reproductive age and has higher rates of emotional difficulties. As women with PCOS tend to have higher androgen levels and androgenic symptoms (e.g., facial hair), we wondered whether women with PCOS symptoms would be less accurate at recognizing emotions than women without PCOS. We examined women with symptoms of PCOS as opposed to only women with PCOS because PCOS is underdiagnosed. Participants were shown a series of faces with different emotions, and were asked to report which emotion was present on each face. The results supported our hypothesis. On the task overall, women with PCOS symptoms were less accurate at recognizing emotions than women without PCOS. When identifying specific emotions, we found that women with high PCOS symptoms made more errors than women without PCOS in recognizing fear and disgust. These results may help us understand more about the role hormones such as androgens may play in emotion recognition, and in conditions like PCOS. The findings also suggest that difficulties with facial emotion recognition may contribute to social and emotional challenges in PCOS.

## Introduction

Polycystic ovary syndrome (PCOS) is the most common endocrine disorder in women of reproductive age and affects 4%–20% of this population.^1^ Several hormones are implicated in the etiology and maintenance of PCOS, including excess androgens.^2^ Symptoms such as hirsutism, acne, and androgenic alopecia are linked to elevated androgen levels.^3^ Research on PCOS can help understand androgenic effects on women’s health and mental health.^1,4,5^

While the etiology of PCOS remains unknown, there is increasing awareness of the psychological and emotional aspects of PCOS.^6,7^ Women with PCOS are more likely to experience emotional dysregulation and mood disorders such as bipolar disorder, depression, and anxiety.^7–11^ These emotional challenges can impact the ability to interpret and respond to social cues, such as facial expressions.^12^

The ability to perceive and identify emotional expressions is called facial emotion recognition (FER). Research indicates that androgens and one’s sex can affect FER abilities. Meta-analyses indicate men are less accurate in FER tasks compared to women.^13^ Further, some studies suggest androgens, such as testosterone (T), may affect FER.^14–21^ However, the gap in understanding how endogenous androgen levels affect FER in women requires further research.

Surprisingly, despite both (a) the emotional and psychological impacts of PCOS and (b) independent lines of research suggesting that androgens impact PCOS and FER, there are no published studies examining FER abilities (e.g., accuracy and reaction time) in women with PCOS. Lai et al. examined PCOS patients at rest and found decreased functional magnetic resonance imaging activity in the left inferior occipital gyrus, a brain region associated with face processing.^22^ However, their study did not include a FER task. Additionally, a study by Dinsdale et al.^23^ examined an adjacent cognitive skill, the ability to perceive complex mental states from only the eyes, in PCOS. While they did not find differences between women with and without PCOS, they did find that women with PCOS taking spironolactone, an androgen receptor antagonist, were more accurate at inferring mental states from images of a person’s eyes than women with PCOS not taking such a medication. Their study examined the perception of complex mental states (e.g., playful and insisting) as opposed to basic emotions, and involved viewing only the eye area (using the Reading the Mind in the Eyes Task) rather than the whole face. However, their findings suggest that androgens may reduce the recognition of basic emotions in women with PCOS. Thus, altered facial emotion processing in PCOS might be one mechanism underlying higher psychological disorders in PCOS.

Two unpublished studies have examined full-face FER in women with PCOS. An unpublished dissertation compared the performance of 54 women without PCOS to 53 women with PCOS.^24^ Participants saw 144 photos of people expressing 1 of 6 different emotions (happy, sad, angry, disgust, fear, and neutral), and were asked to identify which of the 6 emotions was presented in each, as quickly as possible (but participants had an unlimited amount of time to respond). Women with PCOS were less accurate than women without PCOS across all emotions, but particularly in recognizing fear and sad emotions. Another study also reported emotion recognition deficits in women with PCOS.^25^ In this study, 80 women with PCOS and hyperandrogenism were compared to 60 age-matched controls on a FER task using images of actors expressing emotions. Women with PCOS were less accurate in recognizing surprise expressions compared to controls and took longer to identify anger expressions. While these findings suggest subtle FER deficits among women with PCOS, the findings were only reported in abstract form, without full study details.

An important scientific implication of studying facial emotion processing in women with PCOS is that it may clarify how hormones, such as androgens, shape cognitive and socioemotional processing in individuals with and without PCOS. This line of research may also inform emotion perception and regulation models in other populations with hormone-related conditions or hormone use (e.g., menopausal women or transgender individuals undergoing gender-affirming hormone therapy). Clinically, the proposed research could support early identification of women with PCOS who are at risk for mood or social difficulties, and inform targeted interventions that improve emotion regulation and interpersonal functioning.

The present study examined the relationship between PCOS symptoms and FER performance using self-report measures and a FER task. Three groups were compared: women without PCOS, women with provisional PCOS, and men. Hypothesis 1 posited that women would be more accurate than men in the FER task. Hypothesis 2 predicted that women with provisional PCOS would be less accurate than those without PCOS. Hypothesis 3 predicted a positive linear trend such that men would score lower than women with provisional PCOS, and women without PCOS would perform best (i.e., men < women with provisional PCOS < women without PCOS).

## Method

### Participants

The final sample included 178 participants (126 assigned female at birth (AFAB), 52 assigned male at birth) from the university and local community. Nineteen individuals met provisional criteria for a PCOS diagnosis (see below), 107 AFAB individuals did not meet this criterion. Individuals assigned female and male at birth will be referred to as women and men, respectively, in the next sections. The mean age of all participants was 22.64 (SD = 5.91), 71.9% identified as being of White/European descent, and 99% were current university students.

A total of 236 participants initiated the survey. The only initial requirements to participate were: age 16 and older, and access to a computer or device with a stable internet connection. Fifteen participants did not meet the following a priori exclusion criteria: post-menopausal, currently pregnant, currently breastfeeding/lactating, experiencing nicotine withdrawal, or recent alcohol consumption (i.e., any in the 3 h prior to testing or three or more drinks in the prior 8 h). Exclusion criteria applied prior to analyses also excluded participants with: (a) no data on sex (*n* = 27), (b) more than 10% missing data on the FER task and/or outlier scores on happy and sad emotions (*n* = 15), and (c) no data on the exercise covariate data (*n* = 1). Thus, the final sample included 178 participants.

A minimum sample size of 36 (12 per group) was estimated using power package R (v1.3).^26^ Power analyses were computed considering the largest and smallest effect sizes (*d* = 0.58 for sad recognition; 0.40 for fear recognition) from Sukhapure,^24^ a power of 0.80, and an alpha of 0.05.

### Measures

#### Demographics and health questionnaire

This questionnaire collected information on demographics (e.g., age, sex, and ethnicity), health history (e.g., oral contraceptive (OC) use, history of psychological and hormonal disorders), and health behaviors (e.g., alcohol and nicotine use, exercise, and sleep), and these items have been piloted in previous studies.^5,27^ The Positive and Negative Affect Schedule (PANAS)^28^ and the adverse childhood experiences (ACE) questionnaire^29^ were included as potential covariates. Questionnaire items are found in the Supplemental Material Section A: Questionnaire.

#### Polycystic ovary syndrome questionnaire

The polycystic ovary syndrome questionnaire (PCOSQ)^30^ is a four-item screening tool for PCOS that includes items about PCOS symptoms (e.g., length of the menstrual cycle, areas of dark hair growth, obesity, and nipple discharge). It was used to categorize women into those who do (i.e., ⩾2) and those who do not (i.e., <2) meet criteria for provisional PCOS. While this provisional PCOS categorization may include some people with subclinical symptoms, such symptoms impact women’s quality of life^31^ and also require further research. The PCOSQ has a sensitivity of 85% for diagnosing PCOS and a specificity of 93.4%.^30^ As noted below, all participants reporting a diagnosis of PCOS met criteria for provisional PCOS using the PCOSQ, providing validity evidence.

#### Positive and Negative Affect Schedule

The PANAS consists of 20 adjectives that describe affective states, with 10 items for negative affect and 10 items for positive affect.^28^ Participants rated the degree to which they experienced each emotion at the time of testing (i.e., present moment). Response options range from 1 (*very slightly or not at all*) to 5 (*extremely*). Regarding internal consistency, Watson, Clark, and Tellegen reported coefficient alphas for the positive and negative affect subscales of 0.89 and 0.87, respectively.^27^ The PANAS was used to assess participants’ affect and examine group differences, as affect may affect perception of emotion.

#### Adverse childhood experiences questionnaire

The adverse childhood experiences (ACEs) questionnaire^29^ consists of 10 items and assesses 10 types of childhood trauma. Five questions pertain to personal trauma (e.g., physical neglect), and another five questions relate to family trauma (e.g., loss of a parent through death). The ACEs questionnaire has been administered to several populations and there is evidence for retrospective validity as evidenced by agreement among children of the same caregiver.^32^

#### Facial emotion recognition task

The FER task assessed participants’ ability to identify seven basic emotions (anger, disgust, fear, sadness, happiness, surprise, and neutral) at two intensities (low and medium) using 149 images from the Bath intensity variations (ADFES-BIV) database.^33^ Based on our pilot study conducted with 10 participants, a total of 149 images (75 medium intensity, 74 low intensity) were chosen: 23 sad, 23 happy, 23 surprised, 23 fearful, 23 disgusted, 24 angry, and 10 neutral. Participants in the current study identified medium-intensity images with 73% accuracy (SD = 7.95) and low-intensity images with 61% accuracy (SD = 8.43), consistent with Wingenbach et al.^33,34^ Accuracy data were examined in analyses.

The FER task was hosted on the website SurveyMonkey. Participants were presented with an image of an actor emoting anger, disgust, fear, sadness, happiness, surprise, or a neutral expression. They were instructed to use their mouse or keyboard as fast and accurately as possible to identify which emotion they perceived on the face. Responses were either correct, incorrect, or invalid (i.e., no participant response). After completing the FER task, participants provided affective ratings of the emotional faces used. However, that data is not reported here.

### Procedure

This study used an observational case-control design. Recruitment and participation were completed online between February and May 2023. Participants were primarily from Ontario, Canada, and provided written informed consent prior to participating and then completed the above-noted measures (i.e., all questionnaires and the FER task). Female participants were categorized as having provisional PCOS or not having PCOS based on the PCOSQ. The Strengthening the Reporting of Observational Studies in Epidemiology (STROBE) guidelines, listed by Enhancing the Quality and Transparency of health research (EQUATOR) for reporting observational case-control studies, were followed in this report.^35^

#### Statistical analyses

All analyses were completed using IBM SPSS Statistics (IBM Version 28.0.1.1). Data were examined for accuracy, outliers, normality, and homoscedasticity before analyses. If less than 10% of the data were missing within the FER (two items per emotion maximum), mode imputation replaced missing data.^36^ A total of 34 participants’ FER scores required one mode imputation because they were missing less than 10% of data. The same procedure was applied to other scales. Outcome variables from the FER task (i.e., accuracy scores for each of the seven emotions) were examined for outliers (|*z*| ⩾ 3.29) and normality (skewness and kurtosis divided by their standard error <3).^36^ Seven participants were identified as having outliers based on FER scores. Removing outliers normalized happy and sad scores, but surprise and neutral scores exceeded skewness and kurtosis values. Following Tabachnick and Fidell,^36^ these scores were transformed to reduce nonnormality (square root for surprise and log-transformation for neutral scores). Homogeneity tests (Levene’s Test for Equality of Variances and Box’s Test for Equivalence of Covariance Matrices) confirmed that assumptions were met for all analyses.

To test hypotheses 1 and 2, group differences in the total FER scores (i.e., the sum of scores across all emotions) were examined using ANCOVAs, with group as the independent variable. Follow-up Bonferroni-adjusted pairwise comparisons were completed when appropriate. MANCOVAs were run for each hypothesis with seven emotions as the dependent variables (DVs). Significant MANCOVA results (Pillai’s trace *F-*statistic with α < 0.05, trend *p* < 0.07) were followed up with ANCOVAs and pairwise comparisons. Effect sizes were defined as small (~0.01), medium (~0.06), and large (~0.14) using *η_p_*.^2^,^37^ To test hypothesis 3, eight linear contrast analyses tested for linear patterns in FER scores across the three different groups (men < women with provisional PCOS < women without PCOS), with the DVs being the accuracy scores for total FER and each of the seven emotions. To address any potential issues with normality, hypotheses 1 and 2 were also tested using a Quade’s rank analysis of covariance (nonparametric test).

The following variables were considered as potential covariates given links to FER: age, BMI,^38^ alcohol use (i.e., typical frequency of alcohol use, typical number of drinks, and amount of alcohol used in the past 24 h),^39^ hours of sleep last night,^40^ positive affect,^41^ negative affect,^40^ ACE,^42^ ethnicity (White versus Non-White),^43^ OC use,^44^ and amount of exercise in the past 24 h.^45^

## Results

### Validity of PCOS grouping, evaluation of group equivalency, and covariate determination

While 8 of 126 (6.3%) women self-reported a diagnosis of PCOS, 19 of 126 (15.07%) women had a PCOSQ score ⩾2, thereby meeting the criteria for a provisional diagnosis of PCOS.^30^ All women who self-identified as having a PCOS diagnosis also met the criteria for a provisional PCOS diagnosis (100%), providing some validity evidence (i.e., high sensitivity) for the two groups determined by using the PCOSQ cut-off. Women with a provisional PCOS diagnosis differed from those without on both BMI, *F* (1,122) = 34.49, *p* < 0.001 (*M*(SD) = 33.20 (7.32) versus 24.30 (5.54)); and thyroid disorder diagnoses, χ^2^(1, *N* = 123) = 6.66, *p* = 0.010 (11.1% versus 1.0%). These differences provide further validity evidence for the groups, as previous research suggests higher BMI and thyroid disorder rates in PCOS.^46,47^ In terms of the potential covariates, the three groups differed only on the amount of exercise in the prior 24 h, *F* (2,175) = 5.26, *p* = 0.006, and amount of exercise was significantly negatively correlated with recognition accuracy for neutral emotions, *r*(178) = −0.17, *p* = 0.024. Although there were no group differences in OC use (*p* < 0.05), OCs can affect androgen levels,^48,49^ are frequently prescribed to address hormonal issues in women with PCOS,^50^ and are associated with FER.^44^ Therefore, exercise and OC use were both used as covariates in all analyses.

Means and standard deviations for total FER scores and all seven emotions as a function of group are found in Table 1. The results of MANCOVAs, univariate ANCOVAs, linear trend analyses, and all effect sizes for the analyses below are shown in Table 2. Unadjusted analyses had similar outcomes and are found in a table within the Supplemental Material, Section B.

**Table 1.** Unadjusted untransformed means (SDs) for all FER scores as a function of sex and polycystic ovary syndrome groups.

Emotion	Men (*n* = 52)	Women (*n* = 126)	Provisional PCOS (*n* = 19)	No PCOS (*n* = 107)
Total FER	97.69 (9.96)	106.90 (8.75)	102.00 (7.79)	107.78 (8.66)
Sad	15.04 (2.19)	15.67 (2.24)	15.84 (1.86)	15.64 (2.31)
Angry	13.79 (3.54)	15.51 (3.52)	14.95 (3.64)	15.61 (3.51)
Disgust	11.02 (3.85)	13.02 (3.43)	11.32 (3.43)	13.32 (3.36)
Fear	9.52 (4.62)	11.93 (4.31)	9.84 (4.68)	12.30 (4.16)
Happy	18.02 (2.30)	18.84 (2.22)	18.16 (2.27)	18.96 (2.21)
Surprise	19.54 (2.82)	20.84 (2.27)	21.16 (2.24)	20.78 (2.28)
Neutral	9.10 (1.11)	9.33 (0.89)	9.05 (1.03)	9.37 (0.86)

The means here are unadjusted for covariates, but all analyses controlled for amount of exercise in the prior 24 h and oral contraceptive use. Total FER refers to the number of emotional expressions participants identified correctly out of the 149 presented. FER: facial emotion recognition; PCOS: polycystic ovary syndrome; SD: standard deviation.

**Table 2.** Facial emotion recognition MANCOVAs, ANCOVAs, linear trend analyses, and effect sizes.

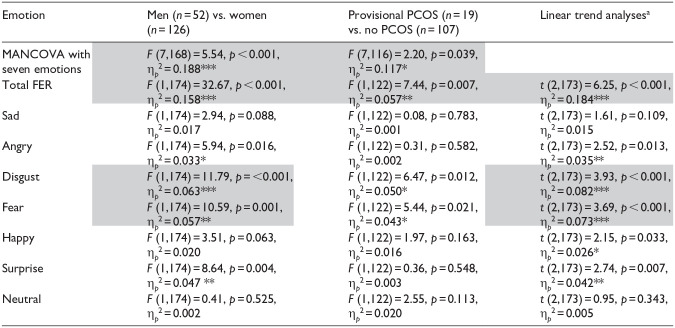

Shaded values reflect medium to large effect sizes. All analyses controlled for amount of exercise in the prior 24 h and oral contraceptive use. PCOS: polycystic ovary syndrome; FER: facial emotion recognition.

a Linear trend analyses test the hypothesis that men < women with provisional PCOS < control women in FER.

* *p* < 0.05. **p < 0.01. ***p < 0.001.

### Hypothesis 1: sex differences

A two-group ANCOVA indicated a significant sex difference, with women being more accurate than men on total FER, *F* (1,174) = 32.67, *p* < 0.001, η_*p*_^2^ *=* 0.158. A MANCOVA with the seven emotions as the DVs also indicated a sex difference with an overall significant multivariate group effect, *F* (7,168) = 5.54, *p* < 0.001, η_*p*_^2^ = 0.188. Follow-up univariate ANCOVAs (see Table 2) indicated that women were significantly more accurate than men with Angry (η_*p*_^2^ = 0.033), Disgust (η_*p*_^2^ = 0.063), Fear (η_*p*_^2^ = 0.057), and Surprise (η_*p*_^2^ = 0.047), recognition. Similar results emerged when using the Quade nonparametric ANCOVAs. Women were significantly more accurate than men with total FER (*t* = −4.93, *p* < 0.001), Angry (*t* = −2.46, *p* = 0.015), Disgust (*t* *=* −3.27, *p* = 0.001), Fear (*t* *=* −3.03, *p* = 0.003), and Surprise (*t* = −2.66, *p* = 0.009) recognition.

The three-group ANCOVA (i.e., men, women without PCOS, women with provisional PCOS) indicated a significant group effect, *F* (2,173) = 20.46, *p* < 0.001, η_*p*_^2^ = 0.191, for total FER. Pairwise comparisons indicated that the sex difference held when men were compared to women without PCOS (i.e., men were less accurate) (*p* < 0.001), but men did not differ from women with provisional PCOS (*p* = 0.086). The findings were the same with Quade’s nonparametric ANCOVAs: a significant sex difference for women without PCOS, *t* = 5.56, *p* < 0.001; and no sex difference when just looking at women with provisional PCOS, *t* = 0.78, *p* = 0.437.

### Hypothesis 2: PCOS group differences

**Figure 1.**
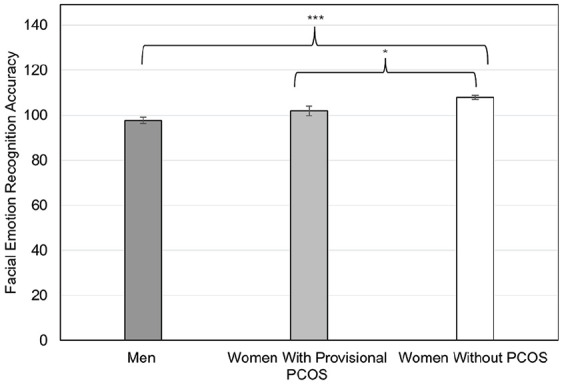
Women with provisional PCOS had lower recognition accuracy on total FER scores than women without provisional PCOS, *F* (1,122) = 7.44, *p* = 0.007, η_*p*_^2^ = 0.057. The sex difference was only found when comparing men with women without provisional PCOS (*p* < 0.001). The linear trend analyses indicated a significant linear effect for total FER scores with men < women with provisional PCOS < control women, *t* (2,173) = 6.25, *p* < 0.001, η_*p*_^2^ = 0.184. Accuracy refers to the total number of images that participants identified correctly out of the 149 images in the task. Error bars reflect standard errors. Means are adjusted for the covariates of exercise and oral contraceptive use. PCOS: polycystic ovary syndrome; FER: facial emotion recognition. *p < 0.05. ***p < 0.001.

A two-group ANCOVA found women with provisional PCOS had significantly lower total FER scores than women without PCOS, *F* (1,122) = 7.44, *p* = 0.007, η_*p*_^2^ = 0.057. This effect is illustrated in Figure 1. The same findings emerged when using Quade’s nonparametric ANCOVA, *t* = 2.89, *p* = 0.005.

**Figure 2.**
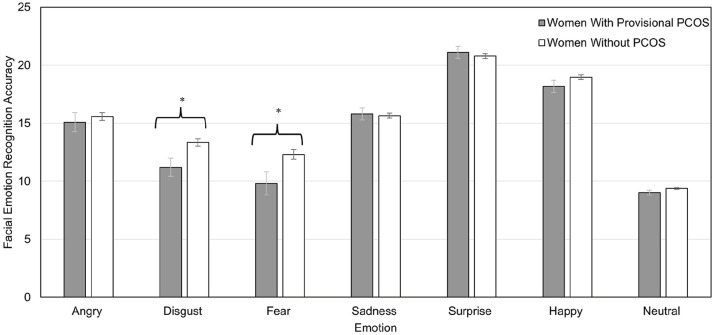
Women with provisional PCOS had significantly lower emotion recognition accuracy scores than women without provisional PCOS for Fear, *F* (1,122) = 5.43, *p* = 0.021, η_*p*_^2^ = 0.043; and Disgust, *F* (1, 122) = 6.47, *p* *=* 0.012, η_*p*_^2^ = 0.050. Significant group differences were not found for the other emotions. Accuracy refers to the total number of images that participants identified correctly out of those presented. Error bars reflect standard errors. Means are adjusted for the covariates of exercise and oral contraceptive use. PCOS: polycystic ovary syndrome. *p < 0.05.

A MANCOVA on the seven individual emotion scores also indicated a significant difference between the two groups, *F* (7,116) = 2.20, *p* = 0.039, η_*p*_^2^ = 0.117. Follow-up ANCOVAs (see Table 2) indicated that women with provisional PCOS were less accurate than women without PCOS in recognizing Fear (η_*p*_^2^ = 0.043) and Disgust (η_*p*_^2^ = 0.050; Figure 2). Similar results were found when using the Quade nonparametric ANCOVAs (i.e., Fear, *t* = 2.29, *p* = 0.023; and Disgust, *t* = 2.44, *p* = 0.016).

### Hypothesis 3: a linear group effect

The results from linear trend analyses suggested a significant positive linear trend across the groups (i.e., men < women with provisional PCOS < women without PCOS) for: Total FER accuracy scores, *t* (2,173) = 6.25, *p* < 0.001, η_*p*_^2^ = 0.184; and Angry (η_*p*_^2^ = 0.035), Disgust (η_*p*_^2^ = 0.082), Fear (η_*p*_^2^ = 0.073), Surprise (η_*p*_^2^ = 0.042), and Happy (η_*p*_^2^ = 0.026) emotions (see Table 2). The pattern for total FER scores is illustrated in Figure 1.

### Supplementary analyses

Given that PCOS symptoms can be viewed dimensionally,^3,30^ supplementary analyses were undertaken to examine the correlations between PCOSQ scores and Total FER, fear, and disgust scores. Spearman correlations controlling for both OC use and exercise revealed a nonsignificant trend for Disgust, *r_s_* (122) = −0.16, *p* = 0.080; a weak nonsignificant trend for total FER, *r_s_* (122) = −0.14, *p* = 0.123; and no association for Fear, *r_s_* (122) = −0.10, *p* = 0.290.

## Discussion

Consistent with hypothesis 1, women were more accurate than men at recognizing facial emotions when all seven emotions were examined together and more accurate at recognizing anger, disgust, fear, surprise, and happy. Hypothesis 2 was also supported, as women with provisional PCOS had significantly lower overall emotion recognition scores compared to women without PCOS, particularly for fearful and disgusted expressions. Hypothesis 3 was also supported, as there were significant linear trend effects for total FER, angry, disgust, fear, surprise, and happy scores. Generally, men were the least accurate, followed by women with provisional PCOS, and women without PCOS were most accurate.

### Hypothesis 1: sex differences and task validity

Women were more accurate than men in detecting emotions (total FER scores) as well as the specific emotions of anger, disgust, fear, surprise, and happy. We did not find a sex difference in detecting neutral emotional expressions. This is consistent with one study that used the same images as those in the present study.^51^ Our finding of a sex difference in overall emotion recognition aligns with the findings of Wingenbach et al.^51^ and Thompson and Voyer’s^13^ meta-analysis in terms of the magnitude of effect size (classified based on Cohen^37^) and directionality.

### Hypotheses 2 and 3: PCOS associated with less accurate facial emotion recognition

The current results suggest that women with provisional PCOS are less accurate at recognizing emotions overall (total FER) and specifically fear and disgust. This fits with research suggesting hormones may affect recognition of disgust^27^ and fear^14^ in women. Less accurate disgust recognition in PCOS is a new finding. It might result in maladaptive food rejection responses and social behavior,^52^ which may have implications for physical and mental health. The finding of reduced emotion recognition accuracy in PCOS is consistent with two unpublished reports.^24,25^ Consistent with our results (in both effect size and directionality), Sukhapure’s^24^ dissertation found that women with PCOS are less accurate at detecting fear (small effect size) than those without the syndrome. While it is unclear whether they examined overall FER scores, the abstract of a study by Czyzyk et al.^25^ indicated that women with PCOS were less accurate in identifying calm and surprise emotions than women without PCOS. The present study is the first full peer-reviewed published report of reduced FER accuracy in PCOS; however, the findings are consistent with these two unpublished studies in terms of suggesting less accurate FER in PCOS. Furthermore, the current findings are consistent with previous research by Dinsdale et al.,^23^ which found that women with PCOS taking an androgen receptor antagonist were better at recognizing mental states when viewing people’s eyes. While their task focused only on the eyes (i.e., not the entire face), and examined more complex mental states (i.e., not basic emotions), their results combined with ours suggest that androgens may have an adverse effect on facial emotion processing in women, and especially those with PCOS.

Given evidence of higher androgen levels in PCOS,^3^ our findings align with studies linking sublingual testosterone (T) administration to altered emotional facial processing in women. The specificity of the present findings to fear is consistent with research suggesting that androgens, such as T may influence the processing of fear-related stimuli. van Honk et al.^20^ reported reduced attention to fearful faces in women given sublingual T during an emotional Stroop task. Similarly, Hermans et al.^53^ reported that women administered T showed a reduced startle reflex to mild electric shocks, as measured by electromyography of facial and eye muscles. Together, these findings suggest that elevated androgen levels may blunt sensitivity to threat-related cues, including the recognition of fear in others. Thus, T may influence emotion recognition in women. However, other factors, such as metabolic issues like insulin dysregulation, may also contribute to cognitive differences in PCOS. For instance, Jarrett et al.^54^ found that poorer performance on mental rotation tasks in women with PCOS was related to hemoglobin levels, not T. Thus, non-androgenic factors might also impact cognitive outcomes in PCOS.^1^

### Limitations and future directions

The present study has three main limitations. First, the study was completed online in a format that did not allow for FER reaction time measurement, resulting in less experimental control than in a lab setting. Nevertheless, Sukhapure^24^ found that women with and without PCOS differed significantly in accuracy but not reaction time, and task validity was demonstrated here by detection of sex differences similar in effect size to previous studies. As well, the overall accuracy rates were in line with previous studies using the ADFES-BIV database. The accuracy rates on the current task were 70% (SD = 10.02), which is consistent with previous research by Wingenbach et al.^51^ who used the same stimuli and noted an overall accuracy rate of 69% (SD = 9.02). Despite these similarities, the lack of standardized administration conditions may have affected the validity of results, possibly even underestimating effect sizes. Future studies could improve data quality by using platforms designed for remote experimental control or by supplementing online tasks with attention or manipulation checks to assess participant engagement. Second, the current study measured provisional PCOS based on self-reported PCOS symptoms as opposed to PCOS diagnosed by a healthcare provider. Both previous studies in the area compared women with and without a clinical diagnosis of PCOS.^24,25^ However, our effect sizes were similar to Sukhapure,^24^ suggesting no issues with power or sensitivity related to sample selection. Third, our sample was recruited primarily from a young adult university population, so generalizing the present findings to the larger population is restricted. The current findings require replication with diverse samples of premenopausal women and also with older menopausal or post-menopausal women with PCOS, because some research suggests that PCOS symptoms can change with age.^1^ Studies should also examine whether emotion recognition is associated with specific biological markers (e.g., androgen levels, androgen genes, and insulin dysregulation) in PCOS, as these may provide insight into biological variables that mediate or moderate the relationship between FER and PCOS symptoms.

### Implications

Poorer emotion recognition, especially lower accuracy in detecting fear, is a deficit that has been associated with mood disorders such as bipolar disorder^55^ and women with PCOS have a greater odds of being diagnosed with mood disorders.^56^ The present findings may help to explain the higher rates of mood disorders and emotional difficulties in women with PCOS, by suggesting emotion recognition deficits as a potential contributor or effect. Understanding these emotional processing difficulties may help explain the higher rates of emotional dysregulation observed in this population. Theoretically, this work highlights the potential role of androgens in shaping sociocognitive and socioemotional processing, particularly in women with hormone-related conditions, and possibly in people taking hormone therapy. Clinically, identifying emotion recognition deficits in women with PCOS may inform early psychotherapeutic or pharmaceutical intervention strategies (e.g., androgen receptor antagonists^23^) aimed at improving emotion regulation and social functioning, and reducing the risk of mood disorders in this group.

## Conclusion

This study suggests that individuals with PCOS or probable PCOS exhibit lower emotion recognition accuracy, including fear and disgust recognition. In quick assessments of emotions, this may lead to unsafe choices. Reduced fear recognition may also hinder opportunities to form social connections. One theory around child-rearing hypothesizes that women are more accurate at recognizing emotions than men due to their primary caregiving roles.^57^ This requires them to be more sensitive to emotional expressions so that they can form connections by responding to their children’s needs. In addition to the adaptive value of fear recognition for parenting, people with lower fear recognition would be less likely to recognize fear (and possibly anxiety) in those around them, thus missing out on an opportunity to comfort and build connections with those individuals. Emotion recognition, especially fear, is also less accurate in adolescent girls with social anxiety^58^ and in mood disorders.^55,59^ Women with PCOS face higher risks for these and other mental health disorders.^56^ Our findings suggest that emotion recognition deficits may contribute to emotional difficulties in the context of PCOS.

## Supplemental Material

sj-doc-1-whe-10.1177_17455057251359761 – Supplemental material for Facial emotion recognition accuracy in women with symptoms of polycystic ovary syndrome: Reduced fear and disgust perception Supplemental material, sj-doc-1-whe-10.1177_17455057251359761 for Facial emotion recognition accuracy in women with symptoms of polycystic ovary syndrome: Reduced fear and disgust perception by Shree Smruthi Venkateshan and Kirsten A. Oinonen in Women’s Health

sj-pdf-2-whe-10.1177_17455057251359761 – Supplemental material for Facial emotion recognition accuracy in women with symptoms of polycystic ovary syndrome: Reduced fear and disgust perception Supplemental material, sj-pdf-2-whe-10.1177_17455057251359761 for Facial emotion recognition accuracy in women with symptoms of polycystic ovary syndrome: Reduced fear and disgust perception by Shree Smruthi Venkateshan and Kirsten A. Oinonen in Women’s Health
