# Supplementary material for: Facial emotion recognition accuracy in women with symptoms of polycystic ovary syndrome: Reduced fear and disgust perception
Source: Womens Health (Lond). 2025 Jul 28;21:17455057251359761. doi: 10.1177/17455057251359761 (PMC12314254; doi:10.1177/17455057251359761)
Supplement: sj-pdf-2-whe-10.1177_17455057251359761 – Supplemental material for Facial emotion recognition accuracy in women with symptoms of polycystic ovary syndrome: Reduced fear and disgust perception [file sj-pdf-2-whe-10.1177_17455057251359761.pdf]

Section A: Questionnaire

The questionnaire below reflects questions included in the study described within Venkateshan and Oinonen's *Women's Health* paper entitled, "Facial Emotion Recognition Accuracy in Women with Symptoms of Polycystic Ovary Syndrome (PCOS): Reduced Fear and Disgust Perception". DOI: 10.1177/17455057251359761

Initial questions make up the *Demographics and Health Questionnaire*.

Note: Some copyrighted measures were removed including images from the Bath Intensity Variations Database (ADFES-BIV),[1,2] Polycystic Ovary Syndrome Questionnaire,[3] Adverse Childhood Experiences Scale,[4] and Positive and Negative Affect Schedule[5]

Initial Questionnaire

Demographics

1. What is your age?
2. What sex were you assigned at birth?
    a. Female
    b. Male
    c. Intersex
    d. Other:
3. What is your current gender identity?
    a. Male
    b. Female
    c. Other (e.g., Two-Spirit) Specify:__________
4. What is your current sexual orientation?
    a. Heterosexual
    b. Gay
    c. Lesbian
    d. Queer
    e. Bisexual
    f. Pansexual
    g. Asexual
    h. Something else that is not already listed here: Specify: ______

5. Please indicate your degree of sexual attraction to women

Not at all attracted to women								Extremely attracted to women
1	2	3	4	5	6	7	8	9

6. Please indicate your degree of sexual attraction to men

Not at all attracted to men								Extremely attracted to men
1	2	3	4	5	6	7	8	9

7. Enter your height in, inches, centimeters, or feet. Use the drop down menu to indicate which measurement you are using (inches, cm, or feet).
   _______ (feet and inches) or _____ (cm)
8. Enter your weight in pounds or kilograms. Use the drop-down menu to indicate which measurement you are using (pounds or kilograms)
   _______ (pounds) or ____ (kg)
9. Please choose the response that represents your ethnic background. Check all that apply.
   a. White, or Euro-American/Canadian
   b. Indigenous
   c. Latin American
   d. Arab
   e. South Asian (e.g., East Indian, Pakistani, Sri Lankan, etc.)
   f. Southeast Asian (e.g., Vietnamese, Cambodian, Laotian, Thai, etc.)
   g. West Asian (e.g., Iranian, Afghan, etc.)
   h. Chinese
   i. Black, Afro-Carribean, or African-American or African-Canadian
   j. Filipino
   k. Korean
   l. Japanese
   m. Other (please specify):
10. What best describes the highest level of education that you have completed?
    a. Some elementary
    b. Completed grade 8
    c. Some high school
    d. Complete high school
    e. Some college
    f. Completed college
    g. Some university
    h. Completed university
    i. Some graduate studies
    j. Completed a graduate degree
11. If you are or were a University/College student what is/was your Major? (e.g., psychology, biology, English, nursing). ___________
12. How many hours of sleep did you get last night? (# hours, 0 - 24 hours) *This question used a drop down menu.*
13. During the past 24 hours how many minutes were you physically active at a moderate to intense level?
    a. 0 minutes
    b. 1 to 15 minutes
    c. 16 to 30 minutes
    d. 31 to 45 minutes
    e. 46 or more minutes
14. Have you had any drinks today (since waking up this morning)?
    a. Yes
    b. No

15. If yes, how many drinks did you consume today (e.g., ONE drink is equal to 1.5 oz distilled alcohol i.e., vodka, rum, whiskey etc., 5 oz glass of wine, or 12 oz bottle of beer).
    Please indicate: ______
16. If you drank alcohol in the past 24 hours, how many hours ago was your last drink? ___ hours
17. What is your typical frequency of alcohol consumption?
    a. Never
    b. Once or twice a month or less
    c. Once or twice a week
    d. Three to four times a week
    e. Almost everyday
18. When you drink alcohol, how many drinks do you typically have on a typical drinking occasion? *(drop down menu with options from 0 to 30+, in increments of 1)*
19. Do you smoke cigarettes, vape, or use other types of nicotine?
    a. Yes
    b. No
20. If you are a smoker or use another form of nicotine, are you currently experiencing nicotine withdrawal (e.g., craving nicotine, feeling angry, irritable, having difficulty concentrating, feeling restless, or anxious)? YES NO MAYBE

Health Information

21. Are you currently taking oral contraceptives (i.e., the birth control pill)?
    a. Yes
    b. No, I have never taken them
    c. No, I used the birth control pill previously and stopped
22. Please check all the types of oral contraceptives you are/were taking. You can check more than one.

Oral Contraceptives:

[ ] Alesse
[ ] Apri
[ ] Aviane
[ ] Brevicon 0.5/35
[ ] Brevicon 1/35
[ ] Cyclen
[ ] Demulen 30
[ ] Loestrin 1.5/30
[ ] Levora
[ ] Marvelon
[ ] MinEstrin 1/20
[ ] Min-Ovral
[ ] Norinyl
[ ] Ortho-Cept
[ ] Ortho 0.5/35
[ ] Ortho 7/7/7
[ ] Ortho 10/11
[ ] Synphasic
[ ] Tri-Cyclen
[ ] Triphasil
[ ] Triquilar
[ ] Demulen 50
[ ] Norlestin 1/50
[ ] Ovral
[ ] Ortho 1/35
[ ] Ortho-Novum 1/50
[ ] Yaz
[ ] Yasmin
[ ] Other: ______

23. Are you currently taking a hormonal contraceptive that is not the oral contraceptive pill (e.g., hormonal IUD, hormonal patch, vaginal rings, injections)?
    a. Yes
    b. No, I have never taken them
    c. No, I used a hormonal contraceptive previously (not including the pill) and stopped
24. Which category best describes your experience with hormonal medication OTHER THAN contraceptives (e.g., hormonal therapy for transitioning, hormone replacement therapy, progestin-only for endometrial cancer, tamoxifen for breast cancer etc.)
    a. Yes, I'm currently using them
    b. Yes, I have used them previously
    c. No, I have never taken them
25. Are you a woman who is currently going through, or has gone through, menopause?
    a. Yes
    b. No
    c. Maybe
26. Are you currently pregnant?
    a. Yes, I'm currently pregnant.
    b. No, I'm not pregnant.
    c. I may be pregnant.
27. Have you ever been pregnant?
    a. Yes, I have been pregnant
    b. No, I have never been pregnant
28. Are you currently breastfeeding?
    a. Yes
    b. No
29. If you have ever attempted to breastfeed following a pregnancy, did you have any difficulties with breast milk supply?

0	1	2	3	4	5
No difficulties					Yes extreme difficulties

30. Have you ever had any head injuries that resulted in permanent changes to your functioning or abilities?
    a. Yes
    b. No
    c. Maybe
31. [Only from women] Please indicate if you ever been diagnosed with or treated for any of the following:

	Yes	No
Depression		
Anxiety		
Bipolar Disorder		
Polycystic Ovary Syndrome		
Diabetes		
Obesity		
Hyper/Hypo Thyroidism		
Cushing's Syndrome		

Acromegaly

32. The Polycystic Ovary Syndrome Questionnaire
Items are not reported for Copyrighted measures. See:

Pedersen SD, Brar S, Faris P, et al. Polycystic ovary syndrome: Validated questionnaire for use in diagnosis. *Can Fam Physician* 2007; 53: 1041–1047.

FER Task Practice Trial Example

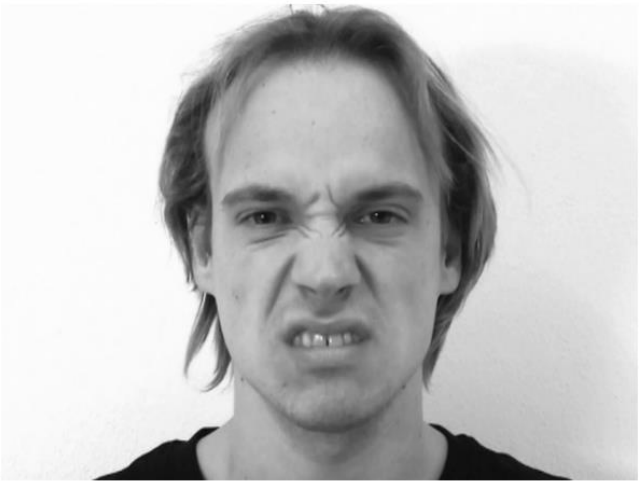

73.

Please select the emotion that is shown in the above image.

sad	angry	disgust	fear	happy	surprise	neutral
○	○	○	○	○	○	○

The image above was retrieved from the Amsterdam Dynamic Facial Expression Set-Bath Intensity Variations database and is included here with permission. For more information, see:

van der Schalk J, Hawk ST, Fischer AH, et al. Moving faces, looking places: Validation of the Amsterdam dynamic facial expression set (ADFES). *Emotion* 2011; 11: 907–920.

Wingenbach TSH, Ashwin C, Brosnan M. Validation of the Amsterdam dynamic facial expression set – bath intensity variations (ADFES-BIV): A set of videos expressing low, intermediate, and high intensity emotions. *PLOS ONE* 2016; 11: e0147112.

33. The Adverse Childhood Experiences Scale

Items are not reported for Copyrighted measures. See:

Felitti VJ, Anda RF, Nordenberg D, et al. Relationship of childhood abuse and household dysfunction to many of the leading causes of death in adults. The adverse childhood experiences (ACE) study. *Am J Prev Med* 1998; 14: 245–258.

34. The Positive and Negative Affect Schedule (PANAS)

Items are not reported for Copyrighted measures. See:

Watson D, Clark LA and Tellegen A. Development and validation of brief measures of positive and negative affect: The PANAS scales. *J Pers Soc Psycholog.* 1988; 54: 1063.

References

1. Van Der Schalk J, Hawk ST, Fischer AH, et al. Moving faces, looking places: validation of the Amsterdam Dynamic Facial Expression Set (ADFES). *Emotion* 2011; 11: 907.

2. Wingenbach TSH, Ashwin C, Brosnan M. Validation of the Amsterdam Dynamic Facial Expression Set – Bath Intensity Variations (ADFES-BIV): A Set of Videos Expressing Low, Intermediate, and High Intensity Emotions. *PLOS ONE* 2016; 11: e0147112.

3. Pedersen SD, Brar S, Faris P, et al. Polycystic ovary syndrome: Validated questionnaire for use in diagnosis. *Can Fam Physician* 2007; 53: 1041–1047.

4. Felitti VJ, Anda RF, Nordenberg D, et al. Relationship of childhood abuse and household dysfunction to many of the leading causes of death in adults. The Adverse Childhood Experiences (ACE) Study. *Am J Prev Med* 1998; 14: 245–258.

5. Watson D, Clark LA, Tellegen A. Development and validation of brief measures of positive and negative affect: the PANAS scales. *J Pers Soc Psychol* 1988; 54: 1063.

Section B

Facial Emotion Recognition MANOVAs, ANOVAs, Linear Trend Analyses, and Effect Sizes

Emotion	Men (n = 52) vs. Women (n = 126)	Provisional PCOS (n = 19) vs. No PCOS (n = 107)	Linear Trend Analyses [a]
MANCOVA with Seven Emotions	F (7,170) = 6.34, p < .001, η_p^2 = .207***	F (7,118) = 2.08, p = .051, η_p^2 = .110	
Total FER	F (1,176) = 37.56, p < .001, η_p^2 = .176***	F (1,124) = 7.38, p = .008, η_p^2 = .056 **	t (2,175) = 6.65, p < .001, η_p^2 = .201 ***
Angry	F (1,176) = 8.75, p = .004, η_p^2 = .047 **	F (1,124) = 0.56, p = .454, η_p^2 = .005	t (2,175) = 3.05, p = .003, η_p^2 = .050 **
Disgust	F (1,176) = 11.61, p < .001, η_p^2 = .062 ***	F (1,124) = 5.71, p = .018, η_p^2 = .044 *	t (2,175) = 3.87, p < .001, η_p^2 = .079***
Fear	F (1,176) = 11.03, p =.001, η_p^2 = .059 ***	F (1,124) = 5.43, p = .021, η_p^2 = .042 *	t (2,175) = 3.78, p < .001, η_p^2 = .075 ***
Sad	F (1,176) = 3.00, p = .085, η_p^2 = .017	F (1,124) = 0.12, p = .726, η_p^2 = .001	t (2,175) = 1.61, p = .110, η_p^2 = .015
Surprise	F (1,176) = 9.91, p = .002, η_p^2 = .053 **	F (1,124) = 0.47, p = .493, η_p^2 = .004	t (2,175) = 2.92, p = .004, η_p^2 = .046 **
Happy	F (1,176) = 4.93, p = .028, η_p^2 = .027 *	F (1,124) = 2.13, p = .147, η_p^2 = .017	t (2,175) = 2.49, p = .014, η_p^2 = .034 *
Neutral	F (1,176) = 2.29, p = .132, η_p^2 = .013	F (1,124) = 2.06, p = .153, η_p^2 = .016	t (2,175) = 1.80, p = .074, η_p^2 = .018

Note: Shaded values reflect medium to large effect sizes. PCOS = Polycystic Ovary Syndrome.
[a] Linear trend analyses test the hypothesis that men < women with provisional PCOS < control women in FER.
*p < 0.05. **p < 0.01. ***p < 0.001
